# Supplementary material for: Predictors of severe strongyloidiasis and mortality in hospitalized patients from Southern Thailand
Source: PLoS Negl Trop Dis. 2026 Apr 20;20(4):e0014252. doi: 10.1371/journal.pntd.0014252 (PMC13108898; doi:10.1371/journal.pntd.0014252)
Supplement: S1 Table — Detailed demographic data, underlying conditions, clinical manifestations, and organ involvement among patients classified as having severe strongyloidiasis. (DOCX) [file pntd.0014252.s001.docx]

**S1 Table.** Detailed Clinical Manifestations and Complications Among Patients with Severe Strongyloidiasis (n = 47)

| **Domain** | **Variable** | **n (%)** |
| --- | --- | --- |
| **Systemic (General)** | Fever | 45 (95.7) |
|  | Chills | 10 (21.3) |
|  | Malaise / Fatigue | 7 (14.9) |
|  | Weight loss | 4 (8.5) |
|  | Mental status change | 12 (25.5) |
|  | Headache | 3 (6.4) |
| **Gastrointestinal** | Any acute GI symptoms | 42 (89.4) |
|  | - Diarrhea | 35 (74.5) |
|  | - Abdominal distension | 11 (23.4) |
|  | - Abdominal pain | 10 (21.3) |
|  | - GI bleeding | 5 (10.6) |
|  | - Ileus / obstruction | 4 (8.5) |
| **Respiratory** | Any acute respiratory symptoms | 26 (55.3) |
|  | - Dyspnea | 23 (48.9) |
|  | - Respiratory failure | 17 (36.2) |
|  | - Cough | 11 (23.4) |
|  | - Hemoptysis | 7 (14.9) |
| **Cutaneous** | Any skin signs | 9 (19.1) |
|  | - Larva currens | 4 (8.5) |
|  | - Petechiae / purpura | 4 (8.5) |
|  | - Maculopapular rash | 1 (2.1) |
| **Strongyloides-related**  **Complications** | Any complication | 22 (46.8) |
|  | - Bacteremia | 18 (38.3) |
|  | - Polymicrobial bacteremia | 4 (8.5) |
|  | - CNS infection (meningitis/brain abscess) ^a^ | 3 (6.4) |
|  | - Septic pulmonary emboli ^b^ | 2 (4.3) |
|  | - Duodenal ulcer or severe mucositis | 3 (6.4) |
|  | - Spontaneous Ileal perforation | 1 (2.1) |
|  | - Abdominal collection | 1 (2.1) |
|  | - Pulmonary hemorrhage | 3 (6.4) |

a) CNS infections included *E. coli* meningitis (n = 1), Enterococcal meningitis (n = 1), and multiple brain abscesses associated with *E. coli* bacteremia (n = 1).
b) Septic pulmonary emboli occurred in patients with *Salmonella* bacteremia (with concordant *Salmonella* spp. growth in sputum culture) (n = 1) and *E. coli* septicemia with multiple brain abscesses (n = 1).
