# Supplementary material for: Predictors of severe strongyloidiasis and mortality in hospitalized patients from Southern Thailand
Source: PLoS Negl Trop Dis. 2026 Apr 20;20(4):e0014252. doi: 10.1371/journal.pntd.0014252 (PMC13108898; doi:10.1371/journal.pntd.0014252)
Supplement: S2 Table — Details of bloodstream infections, including causative organisms, frequency of gram-negative and gram-positive pathogens, and occurrence of polymicrobial infections. (DOCX) [file pntd.0014252.s002.docx]

**S2 Table.** Microbiologic Characteristics of Bacteremia in Severe Strongyloidiasis

| **Category** | **n (%)** |
| --- | --- |
| Monomicrobial bacteremia | 14 (77.8) |
| Polymicrobial bacteremia* | 4 (22.2) |
| Organism (all isolates counted) |  |
| - *Klebsiella pneumoniae* | 10 (43.5) |
| - *Escherichia coli* | 7 (30.4) |
| - *Salmonella* spp. | 3 (13.0) |
| - *Pseudomonas aeruginosa* | 2 (8.7) |
| - *Streptococcus bovis* | 1 (4.3) |

* Polymicrobial bacteremia included the following combinations: P.aeruginosa and K.pneumoniae (n = 1); K.pneumoniae and Salmonella spp. (n = 1); S.bovis, E.coli, and K.pneumoniae (n = 1); and K.pneumoniae, Salmonella spp., and E.coli (n = 1).
