# Supplementary material for: Predictors of severe strongyloidiasis and mortality in hospitalized patients from Southern Thailand
Source: PLoS Negl Trop Dis. 2026 Apr 20;20(4):e0014252. doi: 10.1371/journal.pntd.0014252 (PMC13108898; doi:10.1371/journal.pntd.0014252)
Supplement: S3 Table — Types and frequencies of clinical specimens yielding Strongyloides stercoralis larvae, including stool, respiratory samples, gastrointestinal specimens, and sterile body fluids. (DOCX) [file pntd.0014252.s003.docx]

**S3 Table**. Distribution of Larval Detection by Specimen Type in Severe Strongyloidiasis

| **Specimen** | **Positive, n (%)** | **Negative, n (%)** | **Not performed, n (%)** |
| --- | --- | --- | --- |
| Stool examination | 45 (95.7) | 0 (0) | 2 (4.3) |
| Sputum | 21 (44.7) | 13 (27.7) | 13 (27.7) |
| BAL | 2 (4.3) | 2 (4.3) | 43 (91.5) |
| Gastric aspirate | 3 (6.4) | 10 (21.3) | 34 (72.3) |
| Small bowel aspirate | 2 (4.3) | 0 (0) | 45 (95.7) |
| Pleural fluid | 1 (2.1) | 0 (0) | 46 (97.9) |
| Abdominal collection^a^ | 1 (2.1) | 0 (0) | 46 (97.9) |
| GI biopsy^b^ | 3 (6.4) | 0 (0) | 44 (93.6) |
| Lung biopsy | 1 (2.1) | 0 (0) | 46 (97.9) |

a) An abdominal collection culture grew *Salmonella* spp., suspected to originate from microperforation; however, no perforation was identified on abdominal computed tomography.

b) Gastrointestinal biopsy was performed in patients presenting with gastrointestinal bleeding. Two patients had duodenal ulcers, with histopathology revealing numerous *Strongyloides* organisms. Another patient had severe mucositis extending from the esophagus to the duodenum, and biopsy demonstrated *Strongyloides* within the mucosa.
