# Supplementary material for: Predictors of severe strongyloidiasis and mortality in hospitalized patients from Southern Thailand
Source: PLoS Negl Trop Dis. 2026 Apr 20;20(4):e0014252. doi: 10.1371/journal.pntd.0014252 (PMC13108898; doi:10.1371/journal.pntd.0014252)
Supplement: S4 Table — Summary of chest radiographs findings among patients with severe disease. (DOCX) [file pntd.0014252.s004.docx]

**S4 Table**. Radiographic Findings in Severe Strongyloidiasis with Abnormal Chest Imaging (n = 27)

| **Pattern** | **n (%)** | **Extent of involvement** | **n (%)** |
| --- | --- | --- | --- |
| Patchy opacity | 16 (59.3) | Focal | 9 (33.3) |
| Consolidation | 7 (25.9) | Multifocal | 5 (18.5) |
| Interstitial/reticular infiltration | 5 (18.5) | Diffuse | 13 (48.1) |
| **Zone involved** | **n (%)** | **Pleural effusion** | **n (%)** |
| Upper zone | 6 (22.2) | No effusion | 20 (74.1) |
| Middle zone | 15 (55.6) | Unilateral | 2 (7.4) |
| Lower zone | 21 (77.8) | \| Bilateral \| \| --- \| | 5 (18.5) |
| **Laterality** | **n (%)** | **Pneumothorax** | **n (%)** |
| Right | 5 (18.5) | Presence of pneumothorax | 0 (0) |
| Left | 1 (3.7) |  |  |
| Bilateral | 21 (77.8) |  |  |
