# Supplementary material for: Predictors of severe strongyloidiasis and mortality in hospitalized patients from Southern Thailand
Source: PLoS Negl Trop Dis. 2026 Apr 20;20(4):e0014252. doi: 10.1371/journal.pntd.0014252 (PMC13108898; doi:10.1371/journal.pntd.0014252)
Supplement: Attachment — Submitted filename: Response to reviewers.docx [file pntd.0014252.s007.docx]

**Response to Reviewers**

***Manuscript Title: Predictors of Severe Strongyloidiasis and Mortality in Hospitalized Patients from Southern Thailand***

**Methods:**

**Reviewer #1:**

**Comment 1:** The methods is appropriate for research question.

**Response:** We thank Reviewer #1 for the positive assessment of our methodology.

**Reviewer #2:**

**Comment 1:** The definition of severe strongyloidiasis requiring infectious disease specialist documentation introduces potential ascertainment bias.

**Response:**

We appreciate this important observation. The requirement for infectious diseases specialist documentation was intended to improve diagnostic specificity and reduce misclassification, particularly in patients with complex comorbidities and overlapping clinical syndromes. However, we agree that this criterion may introduce ascertainment bias if specialist consultation rates differed systematically between patient groups.

Importantly, this definition was applied alongside objective microbiologic criteria, specifically the detection of larvae in extraintestinal or respiratory specimens, which are not subject to the same ascertainment concern. In addition, specialist consultation is standard practice for clinically suspected severe infections at our institution, which likely reduces differential ascertainment across groups.

**Manuscript change:** *Added to Limitations (as second limitation): "Second, the severe case definition required infectious diseases specialist documentation, which may introduce ascertainment bias if consultation patterns differed between groups; however, this was applied alongside objective microbiologic evidence and within a setting where specialist consultation is routinely performed for severe infections”*

**Comment 2**. No sample size calculation or power analysis is provided.

**Response:** We acknowledge this point. As this was a retrospective cohort study including all eligible patients over a 16-year period, no a priori sample size calculation was performed. The sample size was determined by the available data. However, the number of outcome events (n = 47 severe cases) was sufficient to support multivariable modelling with an appropriate events-per-variable ratio, thereby reducing the risk of model overfitting.

**Manuscript change:** *Added to Method (Statistical analysis paragraph)*

**Comment 3.** The handling of missing data is not described.

**Response:** We thank the reviewer for this observation. Multivariable models were fitted using complete-case analysis, in which only patients with complete data on all model variables were included. The extent of missing data was low for the key variables (<5%), as laboratory parameters were recorded on the day of diagnosis or within 48 hours per protocol. We have added a statement to the Statistical analysis section of the Methods describing this approach.

**Manuscript change:** *Added to Methods (Statistical analysis paragraph)*

**Comment 4.** Line 160-161 contains an incomplete/redundant sentence ("clinically relevant a priori were included in the multivariable model").

**Response:** We thank the reviewer for identifying this error. The fragment was a duplication that was inadvertently retained during revision. It has been removed from the Methods section.

**Manuscript change:** *Deleted the redundant fragment: "clinically relevant a priori were included in the multivariable model." The surrounding sentence now reads correctly.*

**Comment 5.** Model discrimination (AUC) is mentioned but not reported in results.
**Response:** We apologize for this omission. We have now calculated and reported the AUC values for both multivariable models. The model for severe strongyloidiasis demonstrated moderate discrimination (AUC 0.69), while the 30-day mortality model showed fair-to-good discrimination (AUC 0.74).

**Manuscript change:** *AUC values added to Results following the respective model tables.*

**Results:**

**Reviewer #1:**

**Comment 1:** Corticosteroid exposure - Please provide detail Dose and duration of steroid use. Is the indication of steroid use associate with Strongyloidiasis? Is steroid prescription with other immunosuppressor?

**Respond:**

We thank the reviewer for raising this important point. We attempted to extract detailed information on corticosteroid dose, duration, and indication; however, these variables were not systematically captured in a structured format within the electronic medical records and could not be reliably retrieved for the majority of patients. This is an inherent limitation of the retrospective design and may have resulted in residual confounding, precluding assessment of dose–response relationships.

Regarding co-administration with other immunosuppressive agents, the proportion of patients receiving chemotherapy or other immunosuppressive therapies did not differ significantly between severe and uncomplicated groups (Table 1), suggesting that corticosteroid exposure contributed independently to the observed risk.

**Manuscript change:** *Added to Limitations (as fifth limitation): "Fifth, detailed information on corticosteroid dose, duration, and indication was not systematically captured in the electronic database, precluding dose-response analysis; future studies should prospectively collect these data to better characterize the threshold exposure associated with severe disease."*

**Reviewer #2:**

**Comment 1:** The absence of chronic kidney disease in severe cases (0% vs 10.9%) is unexplained and counterintuitive—this warrants discussion.

**Response:** We thank the reviewer for raising this point. After careful review, we note that CKD is not an established independent risk factor for severe strongyloidiasis. The absence of CKD in the severe group is most parsimoniously explained by the relatively small number of CKD patients in this cohort (n=75) and the low absolute number of severe cases overall (n=47), which together limit the probability of observing CKD in the severe group by chance alone. We have added a brief clarifying statement to the Discussion.

**Manuscript change:** *Added to Discussion (risk factor paragraph): "The lower prevalence of CKD among severe cases (0% vs 10.9%) is likely explained by the relatively small number of CKD patients in this cohort (n=75) and the low absolute number of severe cases overall (n=47), which limits the probability of observing CKD in the severe group by chance alone. CKD is not an established independent risk factor for severe strongyloidiasis, and this finding should not be interpreted as a protective effect."*

**Comment 2.** Model fit statistics and AUC values are not reported despite being mentioned in methods.
**Response:** Please see our response to Reviewer #2, Methods Comment 5 above. AUC values have now been added to the Results section for both models.

**Comment 3.** The indication for stool examination in uncomplicated cases is informative but somewhat tangential to the main analysis.

**Response:** We agree that this section is supplementary to the primary analysis. It was included to contextualise the hospital-based study population and to acknowledge that detection of Strongyloides stercoralis in patients evaluated for diarrhea does not necessarily establish causality. We have retained it as we believe it is of descriptive value, but we are willing to move it to supplementary material if the editor considers this more appropriate.

**Comment 4.** Lack of specific corticosteroid dose exposure
**Response:** Please see our response to Reviewer #1, Results Comment 1 above. Corticosteroid dose and duration data were not available in structured form in our database. This has been explicitly acknowledged as a limitation.

**Conclusions:**

**Reviewer #1:** The conclusion is appropriate.

**Response:** We thank Reviewer #1 for this positive assessment.

**Reviewer #2:**

**Comment 1.** The discussion of HTLV-1 absence, while relevant, is somewhat lengthy given it was not a primary study objective.

**Response:** We agree and have condensed the HTLV-1 discussion to a single brief mention, retaining the relevant references. The revised paragraph acknowledges the absence of HTLV-1 and its consistency with Thailand's epidemiology without elaborating at length.

**Manuscript change:** *Condensed the HTLV-1 passage to: "HTLV-1, a recognized risk factor for severe strongyloidiasis in some endemic regions, was not identified in our cohort, consistent with the very low HTLV-1 prevalence in Thailand."*

**Comment 2.** The clinical implications for screening strategies could be more concrete—what specific recommendations emerge from these findings?

**Response:** We thank the reviewer for this suggestion and have strengthened the clinical recommendations. The revised Discussion now includes a specific, actionable recommendation: clinicians in endemic areas should consider serologic or parasitologic screening for strongyloidiasis in all patients anticipated to initiate corticosteroids or other immunosuppressive therapies, with empiric ivermectin treatment offered if screening cannot be completed prior to treatment initiation.

**Manuscript change:** *Added to Discussion (corticosteroid paragraph): "Based on our findings, clinicians in endemic areas should consider serologic or parasitologic screening for strongyloidiasis in all patients anticipated to initiate corticosteroids or other immunosuppressive therapies, with empiric ivermectin treatment given if screening is not feasible prior to treatment initiation."*

**Comment 3.** The comparison of mortality rates to prior meta-analyses should acknowledge differences in case definitions.
**Response:** We thank the reviewer for this point and agree that the comparison requires qualification. The revised manuscript now includes a note that the meta-analysis used traditional hyperinfection/dissemination criteria, whereas our study applied a pragmatic clinical classification, and that these differences may capture partly different patient populations.

**Manuscript change:** *Revised sentence in Discussion: "The mortality rate among severe cases in our cohort (38.3%) was slightly lower than that reported in a recent meta-analysis (44.8%); however, this comparison should be interpreted cautiously, as that meta-analysis included cases defined under traditional hyperinfection and dissemination criteria, whereas our study applied a pragmatic clinical classification based on multi-organ involvement and specialist documentation, which may capture a somewhat different patient spectrum."*
